# A drug-resistant TB cluster in a refugee family with Mendelian susceptibility to mycobacterial disease (MSDM)

**DOI:** 10.5588/ijtldopen.25.0818

**Published:** 2026-06-15

**Authors:** H.A.Z. Boktor, I. Monedero-Recuero, A. Al Shatnawi, H. Halaseh, M.A. Nejm, S. Satyanarayana, N. Wilson, R. Dent, I. Maaia, K. Okkeh

**Affiliations:** 1 Annoor Sanatorium for Chest Disease, Mafraq, Jordan;; 2 Damien Foundation, Brussels, Belgium;; 3 TB Department, The Union, Paris, France;; 4 International Organisation of Migration, Amman, Jordan;; 5 World Health Organisation, EMRO, Cairo, Egypt;; 6 Directorate of Chest Diseases and Migrant Health, Jordan.

**Keywords:** tuberculosis, Syria, DR-TB, BCG vaccination, MSMD

Dear Editor,

We present a case series for a refugee family, in the context of genetic predisposition,^1^ social vulnerability,^2^ and limited program capacity in complex emergency settings.^3^

## CASE 1

A female child, born in 2015 in a Syrian refugee camp in Jordan, received bacille Calmette-Guerin (BCG) vaccination. Months later, she developed a left axillary lymph node swelling and received several antibiotic courses with no improvement (see Table). Her older brother also received BCG and developed lymph node swelling that was managed with an empiric TB regimen and improved. Initially, the female case was oriented as a locoregional paradoxical inflammation after vaccination (or BCGitis). However, after 1 year, TB was diagnosed with fine-needle aspiration (acid-fast bacilli on microscopy). In June 2016, she began a standard TB regimen, resulting in the resolution of the lymph node swelling. No culture or species identification was available to determine if the source of infection was BCG vaccination (BCGosis, created by *Mycobacterium bovis*) or community-acquired TB.^4,5^

**Table.** Case 1 summary detail of TB episodes.

TB episode	Characteristics	Management	Clinical considerations and course
First episode			
June–December 2016 (1 y/o)	Left axillary lymph nodal TB disease after BCG vaccination	2RHZE/4RH	BCG TB disease versus community-acquired TB disease (bacteriological differentiation not available)
			BCGitis ruled out by AFB positive on lymph node aspiration
			Improvement after TB treatment initiation. Considered cured by clinical improvement
Second episode			
May 2018–March 2019 (3–4 y/o)	TB relapse. Left cervical lymph nodal TB and chest wall mass. Sinus and occasional pus (scrofula)	2RHZ/1RHZE/4RH	BCG disease relapse versus community-acquired new TB infection (bacteriological differentiation not available)
			Regimen inconsistent with international guidelines. Stopped after 10 months. No reported CXR or other tests when declared cured
2019–2020 (4–5 y/o)	Maintained scrofula and systemic symptoms: No limitation in growth and regular development	Close follow-up	Smear, culture, or Xpert not done
			Most likely progressive active TB disease
Third episode			
September 2021 (6 y/o)	Maintained scrofula and worsening of local symptoms	Close follow-up	RR by Xpert on lymph node aspiration. Surgical removal of the lymph node with clinical improvement after weight gain, normalised blood tests, and normal CXR. Referred to an immunologist for evaluation. A genetic immune test was not performed due to financial constraints. Progressive active TB disease (local symptoms, systemic symptoms, and positive Xpert with RR) with no treatment. No culture or species differentiation; most likely community-acquired RR-TB, considering the outbreak pattern affecting other family members
Fourth episode			
August 2022 (7 y/o)	Mild abdominal distention. Clinically worsening. Abdominal ultrasound: severe ascites, enlarged mesenteric lymph nodes, and thrombosis of the portal vein	6Bdq-Dlm-Lvx-Z-E/12Lvx-Z-E	Most likely, this was a continuation of previous episodes initiated in 2018; but not possible to confirm. RR MTBC detected by Xpert: one test RR detected; another RR not detected. Xpert in stools: RR detected
			Treatment initiation after international discussion. Linezolid and Clofazimine were considered but not available in paediatric formulations
			MSMD was diagnosed through the detection of mutations in the IL12RB1 gene
November 2022 (7 y/o)	Clinical improvement	6Bdq-Dlm-Lvx-Z-E/12Lvx-Z-E	Family outbreak investigation: Older brother: diagnosed with MDR/RR-TB and MSMD. Put on the same treatment. Mother: diagnosed with isoniazid-resistant TB. Father: old apical scar in CXR. Grandmother: history of TB decades ago
February 2023 (8 y/o)	5th month of MDR/RR-TB treatment. Recurrent ascites and enlarged mesenteric lymph nodes	Shift into continuation phase (12Lvx-Z-E)	Slow clinical worsening when Bdq and Dlm were stopped. Potential for undiagnosed TB meningitis or disseminated TB
April–May 2023 (8 y/o)	Clinical worsening, severe ascites with hyponatremia. Consciousness lost	Intensive care	Presumed TB affecting the CNS
			Death of the patient in early May 2023

BCG = bacille Calmette-Guerin; Bdq = bedaquiline; CNS = central nervous system; CXR = chest X-ray; Dlm = delamanid; E = ethambutol; H = isoniazid; Lvx = levofloxacin; MDR/RR-TB = multidrug-resistant or rifampicin-resistant TB; MSMD = Mendelian susceptibility to mycobacteria; MTBC = *Mycobacterium tuberculosis complex*; R = rifampicin; RR = rifampicin resistance; Xpert = Xpert MTB/RIF® cartridge; y/o = years old; Z = pyrazinamide.

In March 2018, at 3 years old (y/o), she developed unilateral left cervical lymph node swelling and an anterior chest wall mass. She was diagnosed with TB lymphadenitis relapse (without bacteriological confirmation) and treated with first-line drugs for 10 months. The chest mass subsided, but the lymph node persisted with discharge (scrofula). The treatment was stopped in March 2019 due to a lack of weight gain. It was considered an inflammatory process (BCGitis), and no anti-TB treatment was prescribed. The scrofula remained until 2021. In September 2021 (6 y/o), the lymph node was removed and tested with Xpert MTB/RIF® and culture. Xpert revealed *Mycobacterium tuberculosis complex* (MTBC) with rifampicin resistance (RR). The culture was negative. Differentiating between community-acquired TB and BCG-related TB was again not possible. After surgery, the patient improved clinically (attributable to bacterial burden reduction). An expectant attitude with close follow-up was decided. In August 2022 (7 y/o), she presented abdominal distention and severe underweight (16.4 kg), requiring hospital admission. Ultrasound revealed large ascites, portal vein thrombosis, and multiple enlarged lymph nodes. Peritoneal drainage (800 cc) showed mixed inflammatory cells (no malignancy). Two samples in different laboratories detected *MTBC* by Xpert; one detected RR and the other did not. Other 2 Xpert samples (stool and pulmonary) confirmed *MTBC* with RR, likewise, the peritoneal fluid culture. The patient was enrolled in DR-TB treatment comprising bedaquiline, delamanid, levofloxacin, pyrazinamide, and ethambutol. Linezolid and clofazimine were considered, but paediatric formulations were not available. The parents revealed that they were first cousins, not previously disclosed due to social stigma. A genetic test (whole exome and mitochondrial genome sequencing) showed the presence of a mutation in the IL12RB1 gene (homozygous variant Gln132Ter), confirming Mendelian susceptibility to mycobacterial diseases (MSMD).^6^ She started daily acyclovir, co-trimoxazole, fluconazole, prednisolone, monthly intravenous immune globulin, and daily sub-cutaneous anticoagulant, presenting clinical improvement. In February 2023, there was a 2-kg increase in body weight with mild ascites and unchanged abdominal lymph nodes. In April 2023 (7th month on DR-TB treatment), presented weight loss, ascites, and severe hyponatremia, needing admission and nasogastric feeding. Other MSMD-associated pathogens (including Salmonella spp), viral, and fungal infections were ruled out. She was admitted to a paediatric ICU with presumed central nervous system TB and severe hyponatremia, dying days later. Necropsy was not available.

## CASE 2

In parallel, in 2021, the brother of case 1 (see pedigree tree in the Figure), at 12 y/o, presented a petechial skin rash, painful joint swelling, and lower limb oedema. Chest X-ray (CXR) showed bilateral hilar lymph node enlargement. A skin biopsy revealed leukocytoclastic vasculitis, which is associated with MSMD cases with Salmonellosis and other infections.^7^ No specific treatment was established. In 2022, all previous conditions deteriorated, and in August, considering the clinical circumstances, the previous TB history after BCG, and the sister’s DR-TB and MSDM diagnostics, a presumptive DR-TB diagnosis was established (negative Xpert results). A similar DR-TB regimen as for the sister was initiated, resulting in a quick clinical and laboratory improvement. The same test confirmed MSMD in case 2. There was no financial capacity to screen all family members for MSMD.

**Figure.**
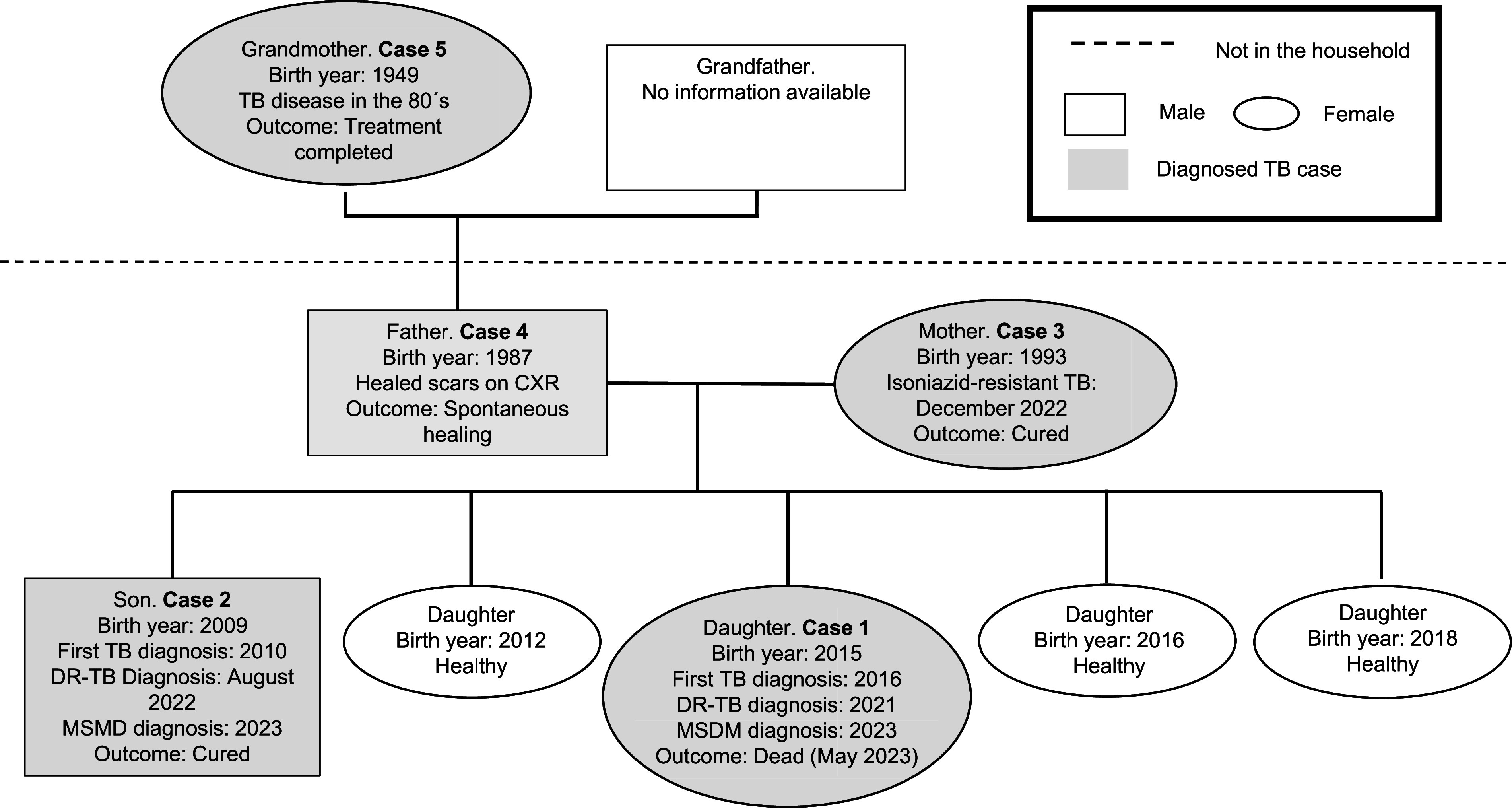
Pedigree tree used for cluster investigation. CXR = chest X-ray; DR-TB = drug-resistant TB; MSMD = Mendelian susceptibility to mycobacterial disease.

## CASES 3–5

In November 2022, all the remaining household members (five individuals) underwent TB screening (symptoms, CXR, sputum smear and culture, Xpert, HIV, and tuberculin skin test). All started levofloxacin TB-preventive therapy in November 2022.^8^ In December 2022, the mother’s sputum culture was positive (case 3 in the cluster); Xpert on the isolates showed *MTBC* detected and RR not detected. Further studies confirmed isoniazid monoresistance. She was treated accordingly and eventually declared cured. The father’s CXR showed a right lung apical region scar. He denied TB disease symptoms but was exposed to TB decades ago by his mother. Father and grandmother are the potential fourth and fifth TB cases in the family, respectively. In this cluster, due to microbiological limitations, the precise disease origins remain undetermined. However, the children were exposed to Mycobacteria from different potential sources: BCG, the mother, the father (CXR with typical TB scars), the grandmother (TB in the past), and the community.

## DISCUSSION

The situation exemplifies the intersection of different vulnerability factors:

1. TB in poor and war-affected communities: The family’s refugee and rampant poverty status highly increases the socio-economic vulnerability, increasing exposure to TB, and limiting access to proper diagnosis and care.^2,3,9^ War and complex emergencies are associated with multiple DR-TB risk factors.^10^
2. Inborn errors of immunity: MSMD has been reported in other Middle East countries and communities with consanguinity. MSMD is a rare genetic disorder (autosomal recessive) caused by mutations in genes involved in the interferon-gamma (IFN-γ) pathway, the most frequent at the IL-12RB1 gene.^6,11,12^ IFN-γ is a key mediator in granuloma development, which is the main host-defence mechanism to contain intra-macrophage micro-organisms like Mycobacteria and Salmonella. Affected individuals are prone to severe and recurrent infections from early childhood. The mortality rate ranges from 40% to 80%.^13^ BCG is contraindicated in patients with MSMD and their newborn siblings until genetic testing is completed.^5^ In addition to antibiotics, recombinant human IFN-γ treatment might be necessary. In extreme cases, stem cell transplantation remains the only option.^5^
3. Limited training and experience in the differential presentation of TB in children: There were important challenges, such as differentiating BCGitis from active TB disease, atypical and bacteriological negative forms of child TB, and a lack of access to spice identification and paediatric formulations. Discordant resistant results, that happen due to different infection sources, resistance acquisition, heteroresistance, low-level resistance, human errors, and others, were particularly confusing in this setting. WHO guidelines enhance the role of contact history, risk factors, clinical presumption, and CXR findings for quick child TB treatment initiation.^14^

In retrospect, this case illustrates important needs in case TB management and household investigation in challenging settings.
